# Precision diagnostics in chronic lymphocytic leukemia: Past, present and future

**DOI:** 10.3389/fonc.2023.1146486

**Published:** 2023-03-21

**Authors:** John Mollstedt, Larry Mansouri, Richard Rosenquist

**Affiliations:** ^1^ Department of Molecular Medicine and Surgery, Karolinska Institutet, Stockholm, Sweden; ^2^ Clinical Genetics, Karolinska University Hospital, Solna, Sweden

**Keywords:** chronic lymphocitic leukemia, next-generation sequencing, genomic aberrations, precision diagnostics, precision medicine

## Abstract

Genetic diagnostics of hematological malignancies has evolved dramatically over the years, from chromosomal banding analysis to next-generation sequencing, with a corresponding increased capacity to detect clinically relevant prognostic and predictive biomarkers. In diagnostics of patients with chronic lymphocytic leukemia (CLL), we currently apply fluorescence *in situ* hybridization (FISH)-based analysis to detect recurrent chromosomal aberrations (del(11q), del(13q), del(17p) and trisomy 12) as well as targeted sequencing (IGHV and *TP53* mutational status) for risk-stratifying purposes. These analyses are performed before start of any line of treatment and assist in clinical decision-making including selection of targeted therapy (BTK and BCL2 inhibitors). Here, we present the current view on the genomic landscape of CLL, including an update on recent advances with potential for clinical translation. We discuss different state-of-the-art technologies that are applied to enable precision diagnostics in CLL and highlight important genomic markers with current prognostic and/or predictive impact as well as those of prospective clinical relevance. In the coming years, it will be important to develop more comprehensive genomic analyses that can capture all types of relevant genetic aberrations, but also to develop highly sensitive assays to detect minor mutations that affect therapy response or confer resistance to targeted therapies. Finally, we will bring up the potential of new technologies and multi-omics analysis to further subclassify the disease and facilitate implementation of precision medicine approaches in this still incurable disease.

## 1 Introduction

The implementation of precision medicine in clinical practice requires a paradigm shift in how health and disease are perceived, from targeting large disease categories, often covering an aggregate of molecular phenotypes that broadly resemble each other in clinical presentation and symptomology, to molecular medicine where the focus is instead placed on selecting targeted therapy for specific molecular alterations based on the patient’s unique molecular features (1). For such specific molecular targeting to be possible, highly sensitive diagnostic methods characterizing the molecular phenotype of the patient is necessary. One such method that has made great advances from bench-to-bedside during the last decade is next-generation sequencing (NGS) or massive parallel sequencing as the technology is also known (2).

Chronic lymphocytic leukemia (CLL) is a disease that is well-positioned at the forefront of these efforts to improve the molecular disease characterization using the new high-throughput sequencing technologies. At the same time, new targeted therapies have been developed and introduced in the treatment of patients with CLL, i.e., BTK and BCL2 inhibitors, hitting the Achille’s heel of the disease and making CLL an ideal candidate for applying the principles of precision medicine (3, 4). CLL is a mature B-cell malignancy characterized by progressively accumulating CD5^+^/CD19^+^ neoplastic B cells in the bone marrow, peripheral blood, and secondary lymphoid organs (5). It is the most commonly occurring leukemia among adults in the Western world, comprising approximately 40% of all leukemias and demonstrating very heterogenous disease courses and manifestations, ranging from asymptomatic disease with no need for therapy, to an aggressive condition demonstrating therapy resistance and short overall survival (OS) (5). While the Rai and Binet clinical staging systems (6, 7) are still used in routine practice to assess prognosis, these cannot identify patients at an early stage, which today constitute the majority of cases (>80%) (8), that will develop a more aggressive disease. Instead, different molecular tests have been introduced with key prognostic and/or predictive impact, including fluorescence *in situ* hybridization (FISH)-based detection of a selected number of chromosomal aberrations, *TP53* gene sequencing, and assessment of the immunoglobulin heavy variable (IGHV) gene somatic hypermutation (SHM) status.

In this review, we discuss clinically relevant genomic aberrations that we currently need to detect in order to risk-stratify and guide treatment selection, including targeted therapy, for patients with CLL, as well as provide our perspective on the current view of the CLL genomic landscape. We will focus on the clinical utility of the different technologies applied, in particular NGS-based techniques, including their strengths and limitations. Finally, we will bring up emerging technologies and how they can further improve molecular profiling and pave the way for the next-generation precision diagnostics in CLL.

## 2 Clinically relevant genomic aberrations in CLL

While the topological details of the genomic landscape of CLL have increasingly emerged with the recent introduction of the new sequencing technologies, certain recurring genomic aberrations, initially demonstrated to be important in the cytogenetic era, are still used in clinical decision-making.

### 2.1 Old facts – the importance of chromosomal aberrations and certain gene mutations

The impact of genetic alterations in CLL was first shown using cytogenetics and chromosome banding analysis in the early 1980’s, where two notable discoveries were the deletion of chromosome 13q [del(13q)] and trisomy 12 (9, 10). Due to the inherent difficulties in culturing CLL cells, FISH analysis was introduced at the turn of the millennium to detect a panel of clinically relevant cytogenetic aberrations i.e., del(11q), del(13q), del(17p), and trisomy 12 (**Table 1**) (14).

**Table 1:** Genetic tests in CLL.

Genetic test	Technology	Frequency	Clinical utility
***TP53* aberrations**	FISH analysis NGS or Sanger	del(17p) and/or *TP53* ^mut^: 5-12%^†^	CIT: Prognostic and predictive BTKi/BCL2i: Predictive
**IGHV gene SHM status**	Sanger or NGS	U-CLL: 30-40% M-CLL: 60-70%	CIT: Prognostic, predictive (M-CLL) BTKi/BLC2i: Predictive (U-CLL)
**Complex** **karyotype**	Cytogenetics or microarrays	High-CK: 5 or more aberrations (2%)^*^	CIT: Prognostic BTKi/BCL2i: To be determined^‡^

^†^(11, 12), ^*^Includes cases without *TP53* aberrations (13). ^‡^Data is still limited for patients treated with BTKi/BCL2 inhibitors. FISH, fluorescence *in situ* hybridization; NGS, next-generation sequencing; CIT, chemoimmunotherapy; BTKi, BTK inhibitor; BCL2i, BCL2 inhibitor.

**Figure 1:**
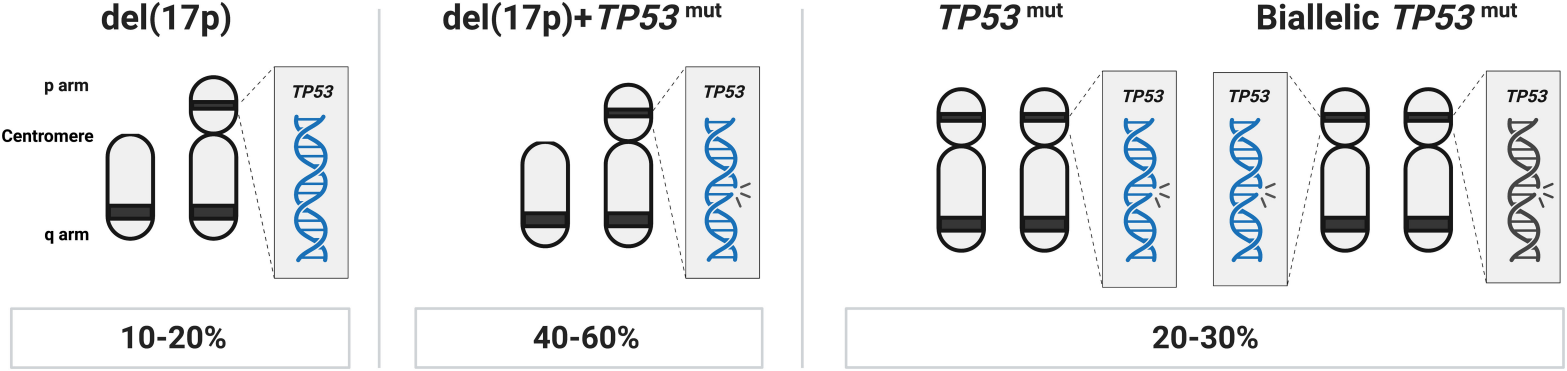
*TP53* abnormalities in CLL. In approximately 40-60% of cases with *TP53* aberrations, del(17p) is found in combination with a *TP53* mutation, another 20-30% of cases carry one or more *TP53* mutations without del(17p), while in the remaining *TP53* aberrant cases (10-20% of patients), only del(17p) is detected.

The del(13q) aberration occurs in more than 50% of cases and in 35 to 40% as the lone genetic aberration. It usually indicates a more favorable prognosis, stable disease, and is associated with the longest time-to-first treatment (TTFT) and OS (14). The minimally deleted region covers two microRNAs, *MIR15A* and *MIR16*, which negatively regulate *BCL2* expression levels (15). del(17p), involving the *TP53* gene, is seen in 5–10% of cases and associated with a rapidly progressing disease, resistance to chemoimmunotherapy, and a very poor clinical outcome (**Table 1**). In roughly 40-60% of cases with *TP53* aberrations, del(17p) is detected together with a *TP53* mutation, while another 20-30% of cases carry one or two *TP53* mutations without del(17p) (**Figure 1**) (11, 16–19). del(11q), involving the *ATM* gene, a tumor suppressor involved in recognizing DNA damage, is found deleted in 10–20% of cases (where a minor proportion (20-25%) of these also carry a second *ATM* mutation) (20) while trisomy 12 is detected in 10-15% of cases. Both of these latter abnormalities are associated with an intermediate prognosis.

In more recent years and owing to improved culturing protocols for cytogenetic analysis, a renewed interest in detecting karyotypic complexity in CLL, defined as five or more chromosomal aberrations, has emerged (**Table 1**) (13). These patients have a particularly poor response to chemoimmunotherapy but also to targeted therapy (13, 21), at least in the relapse/refractory setting. However, analysis of complex karyotype is not generally recommended outside of clinical trials (5).

**Figure 2:**
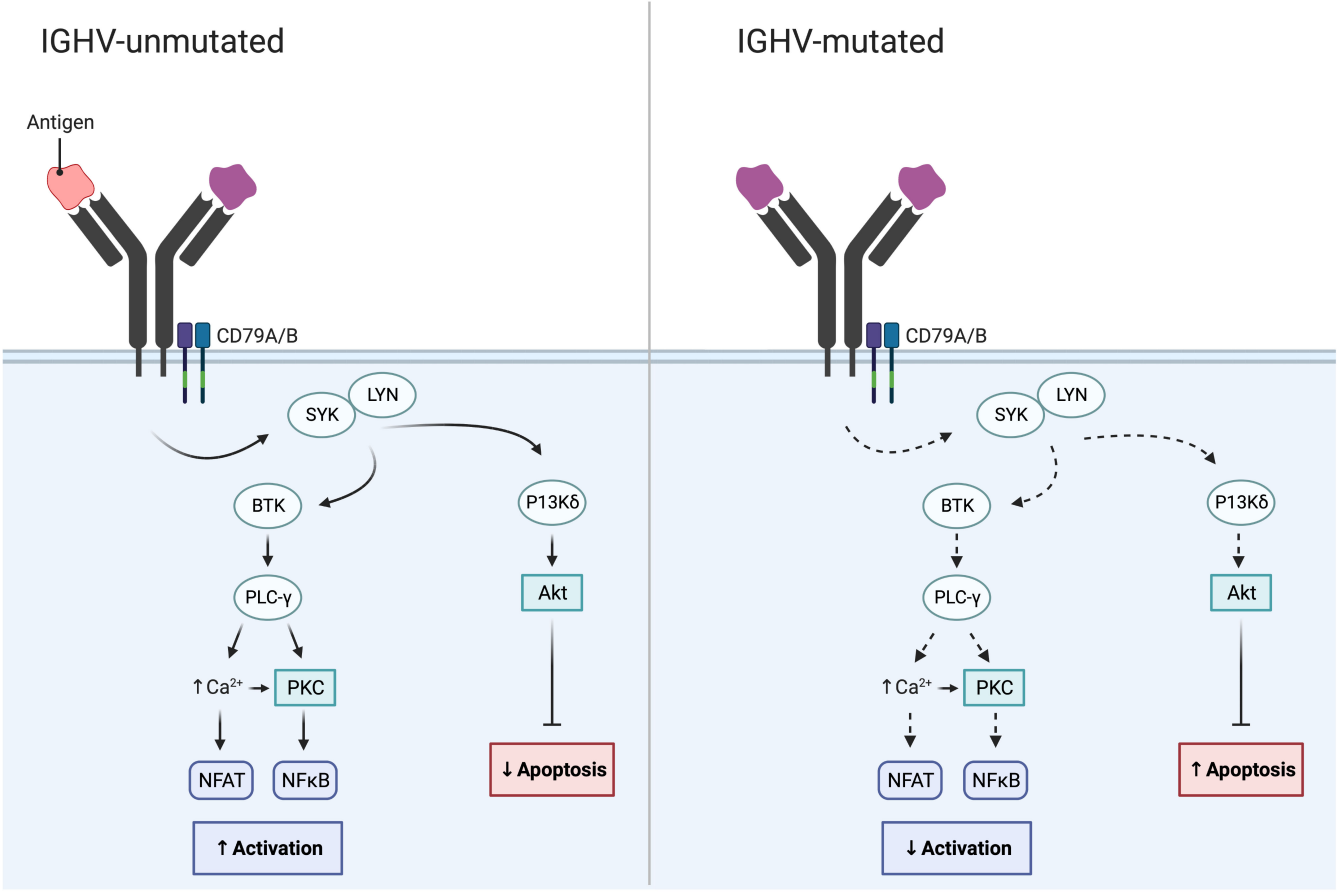
IGHV gene SHM status in CLL and its impact on BcR signaling. While the BcR in IGHV-unmutated CLL often displays polyreactive antigen affinity resulting in increased BcR signaling, the BcR of IGHV-mutated CLL demonstrates reduced BcR signaling and reduced NF-κB activation.

In addition to cytogenetic alterations, a notable and important development was the discovery of the IGHV gene mutational status in 1999; this finding enabled the classification of CLL using the clonotypic SHM status into favorable-prognostic IGHV-mutated (M-CLL; <98% identity to germline) in 60-70% of patients or poor-prognostic IGHV-unmutated (U-CLL; ≥98% identity to germline) in 30-40% of patients (**Table 1**; **Figure 2**) (22, 23). While the IGHV gene mutational status is one of the strongest prognostic markers in CLL, it has also become a predictive marker relevant for therapy selection (discussed further below) (24). Furthermore, it was shown that patients with *TP53* mutations, irrespective of del(17p), have a similarly poor outcome comparable to those with del(17p) (11). Therefore, *TP53* sequencing analysis was added to FISH analysis to detect both types of aberrations (**Table 1**). For both of these molecular tests (IGHV SHM status and *TP53* sequencing), PCR and Sanger sequencing were applied in routine diagnostics to assess their mutational status (11). Sanger sequencing is however a low-throughput and a time-consuming technique, in particular for large genes without hotspot mutations (e.g., *TP53* and *ATM*); these issues were resolved by the introduction of high-throughput NGS-based techniques in the last decade (20, 25). Further underscoring the relevance of *TP53* aberrations and the IGHV gene SHM status, these markers were included in the CLL international prognostic index (CLL-IPI) (26), along with age, β2M levels and stage, which risk-stratifies patients into four categories (low, intermediate, high and very high risk).

### 2.2 The emerging genomic landscape in CLL

NGS technologies utilizing sequencing by synthesis chemistry, where fluorescently labelled dNTPs are sequenced in a massive parallel manner, have enabled the comprehensive characterization of the genomic landscape of CLL. In 2011, by applying whole-exome sequencing (WES) or whole-genome sequencing (WGS), large-scale efforts provided a first detailed molecular map of CLL (27–30). The somatic mutation rate in CLL was estimated to be approximately 0.7 per megabase (this estimate has increased to 1.1 in later studies including larger cohorts), which is comparable to other hematological malignancies, but markedly lower than what can be observed in solid epithelial tumors where the mutation burden is generally observed to be 5–20 times higher than in CLL (28, 31). Important new driver genes included *MYD88, NOTCH1, SF3B1, POT1*, and *XPO1*, and mutations in these genes were associated with clinical outcome (27–30).

**Figure 3:**
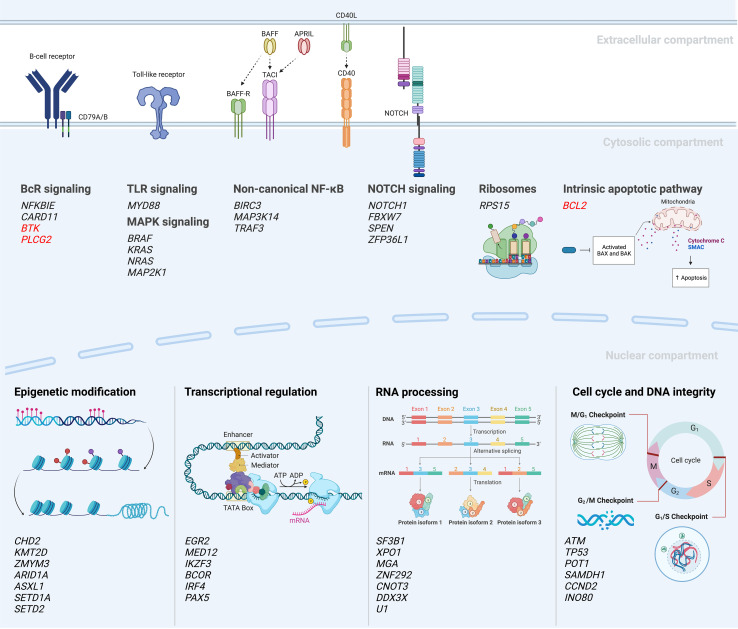
Mutational landscape and deregulated signaling pathways in CLL (18, 27, 30, 32–37). A selection of recurrently mutated genes are included; genes in red indicate mutations emerging during or after targeted therapy.

Following these first descriptions, two seminal papers were published in 2015 which further improved the portrayal of the genomic landscape of CLL, proposing 44 and 59 driver mutations, respectively (32, 33). However, only a few recurrent genomic aberrations (i.e., alterations in *ATM, NOTCH1, SF3B1, TP53*) were observed in more than 10% of cases, followed by a very long tail of hundreds of low-frequency mutated genes present in less than 1-5% of cases. Despite this large heterogeneity, the genomic aberrations could be grouped into more commonly affected signaling pathways and cellular processes, such as B cell receptor (BcR)/NF-κB signaling (*BIRC3, NFKBIE*), NOTCH signaling (*NOTCH1, FBXW7*), DNA repair (*ATM, TP53*), and RNA and ribosome processing (*RPS15*, *SF3B1, XPO1*) (**Figure 3**). The clinical impact of this functional categorization of drivers into cellular pathways was further supported by a study published in 2020 by Brieghel et al, where the number of affected pathways was more important than the number of driver mutations in predicting clinical outcome (38).

The *SF3B1* gene is one of the most frequently mutated genes in CLL, reported to be mutated in 5-15% of early-stage patients with an increasing frequency in relapsed and refractory patients (16-28%) (18, 29, 30, 34, 39–42). Notably, *SF3B1* mutations are particularly enriched in patients belonging to stereotyped subset #2 (up to 45%) (43, 44). SF3B is a complex that forms a functional unit with the U2 small nuclear ribonucleoprotein (snRNP) within the catalytic center of the spliceosome, which is responsible for pre-mRNA processing into mRNA through the excision of introns. The clustering of mutations in hotspot exons (exons 14-16) of the *SF3B1* gene suggests that these mutations have been subject to positive selection and serve as driving events in CLL pathology by giving rise to alternative splicing that affects cellular processes such as DNA damage response, telomere maintenance, and NOTCH signaling (29, 45–48).

Another important discovery was that of *NOTCH1* mutations, which were identified in 8-12% of early-stage patients (18, 27, 28, 34); a number that increases to 13-21% in chemoimmunotherapy refractory patients and above 30% at Richter transformation (28, 41), i.e., progression to a diffuse large B-cell lymphoma with a clinically significantly worse outcome. *NOTCH1* mutations are also enriched in patients with trisomy 12 and in poor-prognostic stereotyped subsets #1 and #8 (44, 49, 50). *NOTCH1* is a transmembrane receptor protein (**Figure 3**) that after activation contributes to the formation of a transcription factor complex consisting of the NOTCH intracellular domain (NCID) and recombinant signal binding protein for immunoglobulin kappa J (RBPJ). A majority of *NOTCH1* mutations (>70%) constitute a 2-base pair frameshift deletion in the PEST domain of the terminal exon, resulting in a premature stop codon and loss of the genetic motive needed for degradation recognition (28, 34, 49). The activity of this transcription factor complex results in the transactivation of downstream target genes, such as *HES1* and *MYC* (49, 51, 52), hence leading to constitutive NF-κB activation (53). Further analysis of the non-coding part of the genome by Puente et al. revealed mutations also in the 3’UTR of *NOTCH1* (32). These non-coding mutations gave an alternative splicing of the PEST domain leading to an increased stability of the protein similar to coding *NOTCH1* mutations.

A further recurrent event resulting in the constitutive activation of the NF-κB pathway in CLL is mutations in *NFKBIE*, which encodes IκBϵ and constitutes one of the negative regulators of the NF-κB pathway (54). *NFKBIE* mutations occur at a frequency of 3-7% in early-stage patients, which rises to 15% in poor-prognostic stereotyped subsets #1, #5 and #6, and are most commonly observed as a 4-bp frameshift deletion leading to a truncated protein and reduced p65 inhibition (34, 35, 54).

*BIRC3* mutations are another recurrent event in CLL with a relatively low frequency at diagnosis (3-4%) (18, 34, 55), but with a higher frequency in fludarabine-refractory patients (up to 24%) (55). BIRC3 contributes to a protein complex that negatively regulates MAP3K14, a central regulatory element in the non-canonical NF-κB pathway (**Figure 3**). Most of the genetic lesions affecting *BIRC3* have been identified as insertions/deletions (indels) resulting in frameshift mutations or premature stop codons located in two hotspot regions between amino acids 367-438 and 537-564 (55, 56).

Additionally, *EGR2*, a transcription factor activated by ERK phosphorylation during BcR signaling, has been found mutated in 2-4% of CLL patients and up to 7-8% in those with advanced-stage disease or Richter transformation (34, 36, 54). *EGR2* is most commonly affected by missense mutations in exon 2. Notably, patients with *EGR2* mutations have a particularly poor outcome, comparable to patients carrying *TP53* aberrations (36).

Finally, a more recently characterized genetic lesion in the pathobiology of CLL is mutations in *RPS15* (ribosomal protein S15), which constitutes a component of the 40S ribsosomal subunit (**Figure 3**). These abnormalities occur at a frequency of 4% in early-stage patients (33, 57) and increase to 20% in patients relapsing following FCR treatment (37). In addition to its role in altering ribosomal fidelity (58, 59), RPS15 may also function as a negative regulator of MDM2-mediated degradation of p53 (37). *RPS15* mutations predominately occur as somatic missense mutations in a 7 amino-acid region of exon 4 and are considered an early clonal event in CLL (37).

### 2.3 Increasing the resolution of the genomic landscape

In a recent study by Knisbacher et al, where WES and WGS data from two previous large efforts were merged and re-analyzed (totaling 1074 CLL cases), the authors could identify 202 candidate genetic drivers in CLL. This included 109 new drivers involving both single nucleotide variants (SNVs), indels and copy-number variants (CNVs) (60). The great majority of patients (>96%) were shown to carry at least one driver lesion, leading to a more complete picture of common pathways hit by genetic lesions, including those involved in genomic stability, chromatin remodeling and ribosomal functioning and biogenesis (**Figure 3**). Examples of novel findings include mutations in *ZFP36L1*, which functions as a tumor suppressor by negatively regulating *NOTCH1* activity, and genetic aberrations in *INO80*, which is involved in genomic stability by coding for the catalytic subunit of a chromatin remodeling complex (60). Additionally, the investigators could also detect genetic lesions shared with other hematological neoplasias, e.g., *RFX7* in diffuse large B-cell lymphoma and Burkitt’s lymphoma as well as the 5q-deletion observed in myelodysplastic syndrome. Finally, the authors noted loss of mitochondrial uncoupling proteins *UCP2* and *UCP3*, caused by del(11)(q13.4), and found in 3% of patients and suggested to act as tumor suppressors by regulating the mitochondrial membrane potential and the efficiency of oxidative phosphorylation (OXPHOS). Importantly, the authors provided further evidence that U-CLL and M-CLL display different genomic landscapes, including significant enrichment of driver mutations in U-CLL compared to M-CLL, with a ratio of approximately 2.8:1 in untreated patients (60). Two examples of genes that were detected as part of this subgroup analysis in U-CLL included *NFKB1*, involved in NF-κB signaling, and *RRM1*, involved in DNA replication and repair and a target of nucleoside analogues such as fludarabine.

In another recent effort, using WGS to characterize 485 CLL patients from the 100 000 Genomes Project in the UK, Robbe and colleagues identified 56 genomic alterations linked to disease outcome, of which 33 were also affected by CNVs and non-coding mutations in regulatory elements. Notably, they detected genetic alterations in 72 promoters, including those associated with known driver genes in CLL, e.g., *BIRC3*, *IKZF3* and *TP53*, as well as the *PAX5* super enhancer region. An example of a novel driver includes del(1)(q42.3), harboring the *IRF2BP2* gene, which could also be affected by SNVs. *IRF2BP2* has previously been implicated in the differentiation of immature B-cells (61). Other examples concern the detection of translocations, where WGS provides a considerable advantage, including t(14;22) with breakpoints in *WDHD1*, indicated to affect translation efficiency in other forms of cancers (62), and the recurrent t(5;6) encompassing *CTNND2* (encodes δ-catenin involved in Wnt signaling) and *ARHGAP18* (involved in cell polarization and migration) in 2-3% of patients (63, 64).

### 2.4 Large-scale validation of recurrent gene alterations

Today, more than 50 genetic lesions have been linked to disease outcome, including *BIRC3*, *EGR2, MYD88, NOTCH1, NFKBIE, POT1*, *RPS15, SETD2, SF3B1, TP53*, and *XPO1* mutations, amongst others (12, 18, 30, 34–37, 49, 55, 65–68). Several retrospective large-scale studies have confirmed the clinical impact of these gene mutations as important prognostic risk factors for both TTFT and OS and proposed different prognostic indices (18, 42, 69). For instance, using recursive partitioning based on OS, Rossi et al. integrated cytogenetic and genetic alterations and proposed four risk groups: i) high-risk harboring *TP53* and/or *BIRC3* aberrations, ii) intermediate-risk with *NOTCH1*, *SF3B1* mutations and/or del(11q), iii) low-risk including trisomy 12 or patients without aberrations, and iv) very low-risk with patients carrying del(13q) (69). In another large-scale study by Baliakas et al. including 3490 patients, sequencing analysis of five genes (*BIRC3, MYD88, NOTCH1, TP53*, and *SF3B1*) showed that *TP53* and *SF3B1* mutations were the strongest factors in multivariate analysis of TTFT (18).

As mentioned, recent studies have also revealed diverse genomic landscapes in M-CLL and U-CLL and indicated that genetic aberrations may affect outcome differently in U-CLL and M-CLL patients (60, 70). To address this issue, Mansouri et al. recently assessed the mutation status of 9 genes (*BIRC3, EGR2, MYD88, NFKBIE, NOTCH1, POT1, SF3B1, TP53*, and *XPO1*) in 4580 patients in relation to the IGHV gene SHM status (34). While *SF3B1* and *XPO1* mutations were strong independent prognostic factors in both U-CLL and M-CLL, *TP53, BIRC3* and *EGR2* alterations predicted outcome only in U-CLL patients and *NOTCH1* and *NFKBIE* in only M-CLL patients (34). These latter findings highlight that all mutations do not confer the same negative impact and that we need a more compartmentalized approach to identify high-risk patients, where genetic aberrations are considered in the context of the IGHV gene SHM status.

Admittedly, most of these retrospective studies included patients treated with chemoimmunotherapy and few prospective studies have been carried out, in particular for patients treated with targeted therapy.

### 2.5 Clonal dynamics and resistance mutations

By combining WES with array-based copy-number analysis, Landau et al. performed in 2013 a detailed investigation of the cancer cell fraction of different CLL-related genomic aberrations. Based on their findings, only a few aberrations were demonstrated to be clonal, i.e., present in the entire cell population (i.e., *MYD88* mutations, del(13q), and trisomy 12), while the vast majority of genomic lesions were present only in a proportion of the cells at subclonal levels (71). Moreover, the clonal dynamics over time was assessed by analyzing samples taken before and after therapy, revealing major clonal shifts in patients relapsing after chemoimmunotherapy. These data have since been confirmed in follow-up studies in larger cohorts treated with chemoimmunotherapy as well as in patients treated with targeted therapy (33, 60, 72).

One such subclonal aberration with potential clinical impact is *TP53* mutations. In a series of papers using deep-sequencing, investigators have detected a significant proportion (2.5-9%) of patients carrying minor subclones with *TP53* mutations (variant allele frequency (VAF) <10%) that were wildtype for *TP53* using Sanger sequencing (73–75). Importantly, patients carrying minor *TP53* mutated subclones appear to have similarly poor outcome as patients with *TP53* mutations detected by Sanger sequencing, at least when treated with chemoimmunotherapy. That said, recent studies based on targeted therapies did not appear to favor selection of *TP53*-aberrant clones in a similar way as chemotherapy (76).

With the introduction of targeted therapies, resistance mutations were detected for patients treated with BTK (e.g., ibrutinib, acalabrutinib) and BCL2 inhibitors (e.g., venetoclax). In patients progressing during treatment with ibrutinib, between 65-90% of patients display *BTK* and/or *PLCG2* mutations (77–79). *BTK* mutations are predominantly seen at the binding site of ibrutinib (amino acid position C481), while mutations of the downstream signaling molecule PLCG2 usually result in a gain-of-function promoting sustained BcR signaling (**Figure 3**). Notably, up to 40% of patients with *BTK/PLCG2* mutations carry minor subclones with VAFs below 10%, or even below 1%, which raises the question as to how these mutations are involved in causing a clinical relapse (77–79). In a proportion of patients relapsing on ibrutinib, other mechanisms have been reported, such as del(8p) (causing loss of TRAIL-R expression) or *BIRC3/NFKBIE* mutations (80). For patients relapsing on venetoclax, *BCL2* mutations have been linked to development of resistance (**Figure 3**) (81) but other mechanisms have also been described, such as upregulation of MCL1 and NOTCH2 (82).

## 3 NGS-based technologies in routine diagnostics

Taking advantage of the versatility of NGS in designing targeted sequencing panels, amplicon-based gene panels including genes recurrently mutated in CLL were rapidly designed. These panels demonstrated a very high correlation with Sanger-detected mutations and provided several orders of magnitude higher sequencing depth compared to Sanger sequencing (20, 25, 83, 84).

To test the reproducibility of NGS-based sequencing, Sutton and colleagues recently performed a comprehensive evaluation of different amplicon-based gene panels targeting 11 CLL-related genes across 6 centers in Europe. Overall, a high (>90%) reproducibility at a VAF cutoff >5% was reported between centers while more heterogenous results were observed below this threshold (84). This shows that amplicon-based NGS can safely be adopted for mutation detection with VAFs >5%, while refinement of methodologies using unique molecular identifiers is necessary to reach a higher sensitivity for the detection of minor variants.

**Figure 4:**
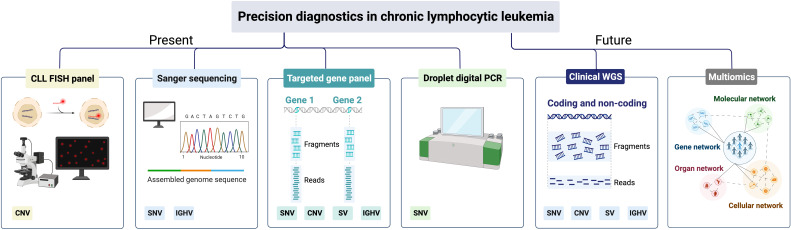
Precision diagnostics in CLL, present status and future directions. SNV, single-nucleotide variants; CNV, copy-number variants; SV, structural variants; IGHV, immunoglobulin heavy variable.

More recently, capture-based gene panels have been introduced that can target a higher number of genes (usually hundreds) with a more uniform sequencing depth, including challenging regions with a high GC content. Another advantage with capture-based panel sequencing is that different types of genomic aberrations, including SNVs/indels, CNVs and structural variants, can be detected simultaneously (**Figure 4**). Using this approach, two capture-based panels were recently validated for lymphoid malignancies including CLL, the Euroclonality-NGS panel and the LYNX panel, which in addition to genomic aberrations (validated for SNVs down to 5% VAF) also assess the IGHV gene SHM status (85, 86).

In today’s precision diagnostics of CLL, we need to be able to detect CNVs affecting *TP53*, i.e., del(17p), and *TP53* mutations in routine diagnostics, since these patients should be considered for targeted therapy (**Table 1**) (87). As *TP53* aberrations appear as subclonal events and may emerge during disease progression and at relapse, FISH analysis and *TP53* sequencing analysis are recommended before start of first treatment and any subsequent line of treatment (5, 19). In many clinical laboratories world-wide, Sanger sequencing has been replaced by amplicon-based or capture-based panel sequencing to assess the *TP53* mutation status (**Figure 4**). To guide laboratories performing *TP53* analysis, the European Research Initiative on CLL (ERIC) has published recommendations as well as introduced an international certification system (19). These recommendations advocate that all coding exons of *TP53* (exons 2-11) should be covered with a recommended sequencing depth of >100x, preferably >500x. Currently, the recommendation is to report pathogenic *TP53* mutations with a VAF above 10% (corresponding to the sensitivity of Sanger sequencing), although mutations in the VAF range 5-10% can be reported if a disclaimer is made that their clinical impact has not been conclusively demonstrated (19). Even though these NGS-based gene panels provide results for the other genes included, they are generally not reported clinically as they will not influence current clinical decision-making but can instead be used for research purposes.

We also need to assess the IGHV gene SHM status as this is both a prognostic and a predictive marker (**Table 1**) (24). The latter aspect has been reinforced with the high clinical efficacy of BTK and/or BCL2 inhibitors in U-CLL patients (88–91). This analysis is recommended to be performed once during the diagnostic process since IGHV SHM status remains constant during the course of the disease. The IGHV gene SHM status is still commonly analyzed by PCR amplification and Sanger sequencing in many clinical laboratories, although new amplicon-based protocols have been published which we anticipate will replace Sanger in the coming years (92). Similar to *TP53* analysis, ERIC has provided detailed recommendations on how to perform and report the IGHV gene SHM status and a system allowing laboratories to certify their analysis (24). In addition, when performing IGHV gene analysis, information on BcR stereotypy can also be retrieved and patients belonging to the poor-prognostic subsets #1, #2 and #8 can be identified (93).

Finally, in the event that a patient progresses after targeted therapy, testing of *BTK, PLCG2* and *BCL2* mutations can be performed using panel sequencing or by performing highly sensitive droplet digital PCR (ddPCR) assays targeting hotspot mutations in subclonal populations (**Figure 4**).

## 4 Discussion

In the last 10 years, NGS-based technologies have been used to characterize the genomic landscape in CLL, which has represented a first critical step in mapping novel genomic biomarkers (27, 30, 32, 33, 60, 94). Based on these studies, a long list of potentially clinically relevant genomic lesions has been proposed, some of which have been validated, albeit commonly in retrospective cohorts (18, 34). In the coming years, it will be essential to validate such potential biomarkers in prospective clinical trials, particularly in relation to targeted therapy, before they can be generally recommended in routine diagnostics. Hopefully, some of the newly discovered disease mechanisms will also enable development of new precision drugs.

Due to the higher sequencing depth, targeted NGS using amplicon or hybrid-capture technologies have rapidly substituted Sanger sequencing in routine diagnostics, usually including a select number of relevant genomic aberrations (**Figure 4**). While WGS has been implemented successfully in rare inherited disease diagnostics, it is also increasingly employed in diagnostics of acute leukemias and pediatric cancers (95, 96). A considerable advantage with WGS is that a complete genomic profile is provided, detecting all types of genomic alterations, e.g., SNVs/indels, CNVs, gene fusions and large structural aberrations, including translocations, at the same time (**Figure 4**). Additionally, WGS can also detect karyotypic complexity and IGHV gene SHM status as well as non-coding mutations e.g., the previously mentioned 3’ UTR mutations in *NOTCH1*, which have been shown to function as driver mutations (32, 94, 97). Finally, having access to a complete genomic profile of a patient prior to start of treatment would also be beneficial for research purposes, e.g., for the detection of novel genetic aberrations linked to disease progression and therapy resistance. Although WGS currently comes at a high cost, the price for sequencing is rapidly decreasing, which may enable full implementation of WGS in the coming years. However, one disadvantage of WGS is the reduced read depth achieved with this technology compared to gene panel sequencing, which limits its usefulness in determining subclonal populations with minor mutations. Hence, we need to continue developing ultra-sensitive deep sequencing or ddPCR protocols, in particular for continuous monitoring of therapeutic response (**Figure 4**).

As the genomic landscape of CLL is emerging, other new technologies are also being investigated, such as transcriptomics, proteomics and drug-response profiling (**Figure 4**). The integration of different omics technologies using multi-omics analytical models has already provided new insights into the molecular landscape of CLL. An example of this is the recent study by Knisbacher and colleagues where they integrated genomic, transcriptomic and epigenomic data into multi-omics models (60). Transcriptomic data identified 8 expression clusters (ECs) with distinctive expression patterns that also correlated with clinical outcome. Additionally, these ECs were strongly associated with epigenetic subtypes based on DNA methylation profiles (i.e., naive-like CLL (n-CLL), intermediate CLL (i-CLL), and memory-like CLL (m-CLL)) (98, 99), IGHV gene SHM status and driver mutations. For instance, del(11q), and *XPO1* and *U1* mutations were enriched in EC-u1, while *SF3B1* and IGLV3-21^R110^ mutations were more frequent in EC-I. In another recent study, the authors integrated genomic, transcriptomic and ATAC-seq data and identified 5 genomic subgroups (GSs) that were linked to certain genomic aberrations, expression signatures and outcome. u-GS1 was characterized by the presence of *TP53* aberrations, short telomeres and MAPK/PI3K mutations, whereas u-GS2 was enriched by *ATM/BIRC3* alterations, and mutations in DNA damage response genes (94, 100).

Yet another example is the recent study by Herbst et al, which integrated genomic, transcriptomic and proteomic data using multi-omics factor analysis (MOFA) combined with drug profiling and discovered 6 subgroups of CLL patients (101). While 5 of these subgroups could be linked to known genetic features (i.e., IGHV gene SHM status, trisomy 12 and *TP53* aberrations), one subgroup was previously unknown and could only be detected when integrating the proteomic data into the analysis. This novel subgroup constituted 20% of patients, was characterized by downregulation of BcR expression, increased splicing, and had a very poor clinical outcome. Previously, MOFA has also been used to identify factors in multi-modal analyses that were not apparent when looking at the data modalities in isolation, highlighting the role of oxidative stress response in CLL (102).

Integration of transcriptomic and proteomic data could potentially also give more immediate insights into the altered phenotype of the patient at the molecular level following introduction of new therapies and therefore contribute to an increased understanding of causality between therapeutic interventions and the resulting molecular phenotype in an unbiased manner. In a recent example by Wang et al, the authors connected transcriptomic and epigenomic data to elucidate the role of BTK inhibition on epigenetic reprogramming (103). Other emerging technologies, such as single-cell sequencing and spatial transcriptomics, have the potential to provide high-resolution insight on the clonal dynamics and evolution of the neoplastic B-cells in CLL and other cells in the tumor microenvironment, which we anticipate will be essential to further our understanding of the disease pathobiology, particularly in regard to development of treatment resistance or Richter transformation (104, 105). In the coming years, using advanced analytical models to reduce complexity and finding patterns in multi-modal data generated from even larger patient cohorts will hopefully pave the way for a more robust subclassification of the disease. It will also be important to develop clinical decision support systems that can assist with data interpretation to identify both prognostic and predictive markers/subgroups as well as integrate various layers of clinicobiological information. Whilst these types of support tools are already an essential part of molecular tumors boards for solid tumors (106), which brings together interdisciplinary teams to discuss individual patient cases, they could also become relevant for CLL in the future to match individual patients to targeted therapy or to ongoing clinical trials.

## 5 Conclusions

Since several decades, we have used prognostic and predictive genetic biomarkers such as FISH-based detection of recurrent chromosomal aberrations and sequenced-based assessment of the *TP53* and IGHV SHM status in the clinical management of CLL. This has greatly increased our ability to risk-stratify patients with CLL and to identify patients that should be treated with targeted therapies. In addition, if a patient does not respond to targeted therapy, we can assess a selected number of genes linked to drug resistance (i.e., *BTK*, *PLCG2* and *BCL2* mutations). In the coming years, multi-omics integration will advance the understanding of the molecular landscape of CLL, where the different data modalities can be used to complement each other, to identify new disease subgroups as well as to provide prognostic and/or predictive markers and new treatment targets. Hopefully, this improved molecular understanding of the disease will enable clinicians and scientists to truly implement precision medicine approaches in the management and treatment of all patients with CLL.

## Author contributions

All authors listed have made a substantial, direct, and intellectual contribution to the work and approved it for publication.

## References

[1] Stenzinger A Edsjo A Ploeger C Friedman M Frohling S Wirta V. Trailblazing precision medicine in Europe: A joint view by genomic medicine Sweden and the centers for personalized medicine, ZPM, in Germany. Semin Cancer Biol (2022) 84:242–54. doi: 10.1016/j.semcancer.2021.05.026 34033893

[2] Walter W Pfarr N Meggendorfer M Jost P Haferlach T Weichert W. Next-generation diagnostics for precision oncology: Preanalytical considerations, technical challenges, and available technologies. Semin Cancer Biol (2022) 84:3–15. doi: 10.1016/j.semcancer.2020.10.015 33171257

[3] Byrd JC Furman RR Coutre SE Flinn IW Burger JA Blum KA. Targeting BTK with ibrutinib in relapsed chronic lymphocytic leukemia. N Engl J Med (2013) 369(1):32–42. doi: 10.1056/NEJMoa1215637 23782158 PMC3772525

[4] Roberts AW Davids MS Pagel JM Kahl BS Puvvada SD Gerecitano JF. Targeting BCL2 with venetoclax in relapsed chronic lymphocytic leukemia. N Engl J Med (2016) 374(4):311–22. doi: 10.1056/NEJMoa1513257 PMC7107002 26639348

[5] Hallek M Cheson BD Catovsky D Caligaris-Cappio F Dighiero G Dohner H. iwCLL guidelines for diagnosis, indications for treatment, response assessment, and supportive management of CLL. Blood (2018) 131(25):2745–60. doi: 10.1182/blood-2017-09-806398 29540348

[6] Rai KR Sawitsky A Cronkite EP Chanana AD Levy RN Pasternack BS. Clinical staging of chronic lymphocytic leukemia. Blood (1975) 46(2):219–34. doi: 10.1182/blood.V46.2.219.219 27789434

[7] Binet JL Lepoprier M Dighiero G Charron D D'Athis P Vaugier G. A clinical staging system for chronic lymphocytic leukemia: prognostic significance. Cancer (1977) 40(2):855–64. doi: 10.1002/1097-0142(197708)40:2<855::AID-CNCR2820400239>3.0.CO;2-1 890666

[8] Baliakas P Mattsson M Stamatopoulos K Rosenquist R. Prognostic indices in chronic lymphocytic leukaemia: Where do we stand how do we proceed? J Intern Med (2016) 279(4):347–57. doi: 10.1111/joim.12455 26709197

[9] Gahrton G Robert KH Friberg K Zech L Bird AG. Extra chromosome 12 in chronic lymphocytic leukaemia. Lancet (1980) 1(8160):146–7. doi: 10.1016/S0140-6736(80)90622-4 6101473

[10] Zech L Mellstedt H. Chromosome 13–a new marker for b-cell chronic lymphocytic leukemia. Hereditas (1988) 108(1):77–84. doi: 10.1111/j.1601-5223.1988.tb00684.x 3259569

[11] Campo E Cymbalista F Ghia P Jager U Pospisilova S Rosenquist R. TP53 aberrations in chronic lymphocytic leukemia: An overview of the clinical implications of improved diagnostics. Haematologica (2018) 103(12):1956–68. doi: 10.3324/haematol.2018.187583 PMC6269313 30442727

[12] Zenz T Eichhorst B Busch R Denzel T Habe S Winkler D. TP53 mutation and survival in chronic lymphocytic leukemia. J Clin Oncol (2010) 28(29):4473–9. doi: 10.1200/JCO.2009.27.8762 20697090

[13] Baliakas P Jeromin S Iskas M Puiggros A Plevova K Nguyen-Khac F. Cytogenetic complexity in chronic lymphocytic leukemia: definitions, associations, and clinical impact. Blood (2019) 133(11):1205–16. doi: 10.1182/blood-2018-09-873083 PMC6509568 30602617

[14] Dohner H Stilgenbauer S Benner A Leupolt E Krober A Bullinger L. Genomic aberrations and survival in chronic lymphocytic leukemia. N Engl J Med (2000) 343(26):1910–6. doi: 10.1056/NEJM200012283432602 11136261

[15] Cimmino A Calin GA Fabbri M Iorio MV Ferracin M Shimizu M. miR-15 and miR-16 induce apoptosis by targeting BCL2. Proc Natl Acad Sci U.S.A. (2005) 102(39):13944–9. doi: 10.1073/pnas.0506654102 PMC1236577 16166262

[16] Dicker F Herholz H Schnittger S Nakao A Patten N Wu L. The detection of TP53 mutations in chronic lymphocytic leukemia independently predicts rapid disease progression and is highly correlated with a complex aberrant karyotype. Leukemia (2009) 23(1):117–24. doi: 10.1038/leu.2008.274 18843282

[17] Rossi D Cerri M Deambrogi C Sozzi E Cresta S Rasi S. The prognostic value of TP53 mutations in chronic lymphocytic leukemia is independent of Del17p13: implications for overall survival and chemorefractoriness. Clin Cancer Res (2009) 15(3):995–1004. doi: 10.1158/1078-0432.CCR-08-1630 19188171

[18] Baliakas P Hadzidimitriou A Sutton LA Rossi D Minga E Villamor N. Recurrent mutations refine prognosis in chronic lymphocytic leukemia. Leukemia (2015) 29(2):329–36. doi: 10.1038/leu.2014.196 24943832

[19] Malcikova J Tausch E Rossi D Sutton LA Soussi T Zenz T. ERIC recommendations for TP53 mutation analysis in chronic lymphocytic leukemia-update on methodological approaches and results interpretation. Leukemia (2018) 32(5):1070–80. doi: 10.1038/s41375-017-0007-7 PMC5940638 29467486

[20] Mansouri L Sutton LA Ljungstrom V Sorqvist EF Gunnarsson R Smedby KE. Feasibility of targeted next-generation sequencing of the TP53 and ATM genes in chronic lymphocytic leukemia. Leukemia (2014) 28(3):694–6. doi: 10.1038/leu.2013.322 24172824

[21] Baliakas P Espinet B Mellink C Jarosova M Athanasiadou A Ghia P. Cytogenetics in chronic lymphocytic leukemia: ERIC perspectives and recommendations. Hemasphere (2022) 6(4):e707. doi: 10.1097/HS9.0000000000000707 35392482 PMC8984316

[22] Hamblin TJ Davis Z Gardiner A Oscier DG Stevenson FK. Unmutated ig V(H) genes are associated with a more aggressive form of chronic lymphocytic leukemia. Blood (1999) 94(6):1848–54. doi: 10.1182/blood.V94.6.1848 10477713

[23] Damle RN Wasil T Fais F Ghiotto F Valetto A Allen SL. Ig V gene mutation status and CD38 expression as novel prognostic indicators in chronic lymphocytic leukemia. Blood (1999) 94(6):1840–7. doi: 10.1182/blood.V94.6.1840 10477712

[24] Agathangelidis A Chatzidimitriou A Chatzikonstantinou T Tresoldi C Davis Z Giudicelli V. Immunoglobulin gene sequence analysis in chronic lymphocytic leukemia: the 2022 update of the recommendations by ERIC, the European research initiative on CLL. Leukemia (2022) 36(8):1961–8. doi: 10.1038/s41375-022-01604-2 PMC9343247 35614318

[25] Sutton LA Ljungstrom V Mansouri L Young E Cortese D Navrkalova V. Targeted next-generation sequencing in chronic lymphocytic leukemia: A high-throughput yet tailored approach will facilitate implementation in a clinical setting. Haematologica (2015) 100(3):370–6. doi: 10.3324/haematol.2014.109777 PMC4349276 25480502

[26] Pflug N Bahlo J Shanafelt TD Eichhorst BF Bergmann MA Elter T. Development of a comprehensive prognostic index for patients with chronic lymphocytic leukemia. Blood (2014) 124(1):49–62. doi: 10.1182/blood-2014-02-556399 24797299 PMC4260976

[27] Puente XS Pinyol M Quesada V Conde L Ordonez GR Villamor N. Whole-genome sequencing identifies recurrent mutations in chronic lymphocytic leukaemia. Nature (2011) 475(7354):101–5. doi: 10.1038/nature10113 PMC3322590 21642962

[28] Fabbri G Rasi S Rossi D Trifonov V Khiabanian H Ma J. Analysis of the chronic lymphocytic leukemia coding genome: role of NOTCH1 mutational activation. J Exp Med (2011) 208(7):1389–401. doi: 10.1084/jem.20110921 PMC3135373 21670202

[29] Wang L Lawrence MS Wan Y Stojanov P Sougnez C Stevenson K. SF3B1 and other novel cancer genes in chronic lymphocytic leukemia. N Engl J Med (2011) 365(26):2497–506. doi: 10.1056/NEJMoa1109016 PMC3685413 22150006

[30] Quesada V Conde L Villamor N Ordonez GR Jares P Bassaganyas L. Exome sequencing identifies recurrent mutations of the splicing factor SF3B1 gene in chronic lymphocytic leukemia. Nat Genet (2012) 44(1):47–52. doi: 10.1038/ng.1032 22158541

[31] Alexandrov LB Nik-Zainal S Wedge DC Aparicio SA Behjati S Biankin AV. Signatures of mutational processes in human cancer. Nature (2013) 500(7463):415–21. doi: 10.1038/nature12477 PMC3776390 23945592

[32] Puente XS Bea S Valdes-Mas R Villamor N Gutierrez-Abril J Martin-Subero JI. Non-coding recurrent mutations in chronic lymphocytic leukaemia. Nature (2015) 526(7574):519–24. doi: 10.1038/nature14666 26200345

[33] Landau DA Tausch E Taylor-Weiner AN Stewart C Reiter JG Bahlo J. Mutations driving CLL and their evolution in progression and relapse. Nature (2015) 526(7574):525–30. doi: 10.1038/nature15395 PMC4815041 26466571

[34] Mansouri L Thorvaldsdottir B Sutton LA Karakatsoulis G Meggendorfer M Parker H. Different prognostic impact of recurrent gene mutations in chronic lymphocytic leukemia depending on IGHV gene somatic hypermutation status: A study by ERIC in HARMONY. Leukemia (2022) 37(2):339–347. doi: 10.1038/s41375-022-01802-y PMC9898037 36566271

[35] Mansouri L Sutton LA Ljungstrom V Bondza S Arngarden L Bhoi S. Functional loss of IkappaBepsilon leads to NF-kappaB deregulation in aggressive chronic lymphocytic leukemia. J Exp Med (2015) 212(6):833–43. doi: 10.1084/jem.20142009 PMC4451125 25987724

[36] Young E Noerenberg D Mansouri L Ljungstrom V Frick M Sutton LA. EGR2 mutations define a new clinically aggressive subgroup of chronic lymphocytic leukemia. Leukemia (2017) 31(7):1547–54. doi: 10.1038/leu.2016.359 PMC7001738 27890934

[37] Ljungstrom V Cortese D Young E Pandzic T Mansouri L Plevova K. Whole-exome sequencing in relapsing chronic lymphocytic leukemia: Clinical impact of recurrent RPS15 mutations. Blood (2016) 127(8):1007–16. doi: 10.1182/blood-2015-10-674572 PMC4768426 26675346

[38] Brieghel C da Cunha-Bang C Yde CW Schmidt AY Kinalis S Nadeu F. The number of signaling pathways altered by driver mutations in chronic lymphocytic leukemia impacts disease outcome. Clin Cancer Res (2020) 26(6):1507–15. doi: 10.1158/1078-0432.CCR-18-4158 31919133

[39] Guieze R Robbe P Clifford R de Guibert S Pereira B Timbs A. Presence of multiple recurrent mutations confers poor trial outcome of relapsed/refractory CLL. Blood (2015) 126(18):2110–7. doi: 10.1182/blood-2015-05-647578 26316624

[40] Rossi D Bruscaggin A Spina V Rasi S Khiabanian H Messina M. Mutations of the SF3B1 splicing factor in chronic lymphocytic leukemia: Association with progression and fludarabine-refractoriness. Blood (2011) 118(26):6904–8. doi: 10.1182/blood-2011-08-373159 PMC3245210 22039264

[41] Schnaiter A Paschka P Rossi M Zenz T Buhler A Winkler D. NOTCH1, SF3B1, and TP53 mutations in fludarabine-refractory CLL patients treated with alemtuzumab: Results from the CLL2H trial of the GCLLSG. Blood (2013) 122(7):1266–70. doi: 10.1182/blood-2013-03-488197 23821658

[42] Jeromin S Weissmann S Haferlach C Dicker F Bayer K Grossmann V. SF3B1 mutations correlated to cytogenetics and mutations in NOTCH1, FBXW7, MYD88, XPO1 and TP53 in 1160 untreated CLL patients. Leukemia (2013) 28(1):108–17. doi: 10.1038/leu.2013.263 24113472

[43] Strefford JC Sutton LA Baliakas P Agathangelidis A Malcikova J Plevova K. Distinct patterns of novel gene mutations in poor-prognostic stereotyped subsets of chronic lymphocytic leukemia: The case of SF3B1 and subset 2. Leukemia (2013) 27(11):2196–9. doi: 10.1038/leu.2013.98 23558524

[44] Sutton LA Young E Baliakas P Hadzidimitriou A Moysiadis T Plevova K. Different spectra of recurrent gene mutations in subsets of chronic lymphocytic leukemia harboring stereotyped b-cell receptors. Haematologica (2016) 101(8):959–67. doi: 10.3324/haematol.2016.141812 PMC4967575 27198719

[45] Furney SJ Pedersen M Gentien D Dumont AG Rapinat A Desjardins L. SF3B1 mutations are associated with alternative splicing in uveal melanoma. Cancer Discovery (2013) 3(10):1122–9. doi: 10.1158/2159-8290.CD-13-0330 PMC5321577 23861464

[46] Te Raa GD Derks IA Navrkalova V Skowronska A Moerland PD van Laar J. The impact of SF3B1 mutations in CLL on the DNA-damage response. Leukemia (2015) 29(5):1133–42. doi: 10.1038/leu.2014.318 25371178

[47] Wang L Brooks AN Fan J Wan Y Gambe R Li S. Transcriptomic characterization of SF3B1 mutation reveals its pleiotropic effects in chronic lymphocytic leukemia. Cancer Cell (2016) 30(5):750–63. doi: 10.1016/j.ccell.2016.10.005 PMC5127278 27818134

[48] Leeksma AC Derks IAM Kasem MH Kilic E de Klein A Jager MJ. The effect of SF3B1 mutation on the DNA damage response and nonsense-mediated mRNA decay in cancer. Front Oncol (2020) 10:609409. doi: 10.3389/fonc.2020.609409 33585229 PMC7880055

[49] Rossi D Rasi S Fabbri G Spina V Fangazio M Forconi F. Mutations of NOTCH1 are an independent predictor of survival in chronic lymphocytic leukemia. Blood (2012) 119(2):521–9. doi: 10.1182/blood-2011-09-379966 PMC3257017 22077063

[50] Balatti V Bottoni A Palamarchuk A Alder H Rassenti LZ Kipps TJ. NOTCH1 mutations in CLL associated with trisomy 12. Blood (2012) 119(2):329–31. doi: 10.1182/blood-2011-10-386144 PMC3257004 22086416

[51] Jarriault S Brou C Logeat F Schroeter EH Kopan R Israel A. Signalling downstream of activated mammalian notch. Nature (1995) 377(6547):355–8. doi: 10.1038/377355a0 7566092

[52] Satoh Y Matsumura I Tanaka H Ezoe S Sugahara H Mizuki M. Roles for c-myc in self-renewal of hematopoietic stem cells. J Biol Chem (2004) 279(24):24986–93. doi: 10.1074/jbc.M400407200 15067010

[53] Rosati E Sabatini R Rampino G Tabilio A Di Ianni M Fettucciari K. Constitutively activated notch signaling is involved in survival and apoptosis resistance of b-CLL cells. Blood (2009) 113(4):856–65. doi: 10.1182/blood-2008-02-139725 18796623

[54] Damm F Mylonas E Cosson A Yoshida K Della Valle V Mouly E. Acquired initiating mutations in early hematopoietic cells of CLL patients. Cancer Discovery (2014) 4(9):1088–101. doi: 10.1158/2159-8290.CD-14-0104 24920063

[55] Rossi D Fangazio M Rasi S Vaisitti T Monti S Cresta S. Disruption of BIRC3 associates with fludarabine chemorefractoriness in TP53 wild-type chronic lymphocytic leukemia. Blood (2012) 119(12):2854–62. doi: 10.1182/blood-2011-12-395673 22308293

[56] Diop F Moia R Favini C Spaccarotella E De Paoli L Bruscaggin A. Biological and clinical implications of BIRC3 mutations in chronic lymphocytic leukemia. Haematologica (2020) 105(2):448–56. doi: 10.3324/haematol.2019.219550 PMC7012473 31371416

[57] Tausch E Beck P Schlenk RF Jebaraj BJ Dolnik A Yosifov DY. Prognostic and predictive role of gene mutations in chronic lymphocytic leukemia: results from the pivotal phase III study COMPLEMENT1. Haematologica (2020) 105(10):2440–7. doi: 10.3324/haematol.2019.229161 PMC7556677 33054084

[58] Bretones G Alvarez MG Arango JR Rodriguez D Nadeu F Prado MA. Altered patterns of global protein synthesis and translational fidelity in RPS15-mutated chronic lymphocytic leukemia. Blood (2018) 132(22):2375–88. doi: 10.1182/blood-2017-09-804401 PMC6410914 30181176

[59] Ntoufa S Gerousi M Laidou S Psomopoulos F Tsiolas G Moysiadis T. RPS15 mutations rewire RNA translation in chronic lymphocytic leukemia. Blood Adv (2021) 5(13):2788–92. doi: 10.1182/bloodadvances.2020001717 PMC8288675 34251413

[60] Knisbacher BA Lin Z Hahn CK Nadeu F Duran-Ferrer M Stevenson KE. Molecular map of chronic lymphocytic leukemia and its impact on outcome. Nat Genet (2022) 54(11):1664–1674. doi: 10.1038/s41588-022-01140-w PMC10084830 35927489

[61] Keller MD Pandey R Li D Glessner J Tian L Henrickson SE. Mutation in IRF2BP2 is responsible for a familial form of common variable immunodeficiency disorder. J Allergy Clin Immunol (2016) 138(2):544–50.e4. doi: 10.1016/j.jaci.2016.01.018 27016798 PMC4976039

[62] Ertay A Liu H Liu D Peng P Hill C Xiong H. WDHD1 is essential for the survival of PTEN-inactive triple-negative breast cancer. Cell Death Dis (2020) 11(11):1001. doi: 10.1038/s41419-020-03210-5 33221821 PMC7680459

[63] Huang F Chen J Wang Z Lan R Fu L Zhang L. Delta-catenin promotes tumorigenesis and metastasis of lung adenocarcinoma. Oncol Rep (2018) 39(2):809–17. doi: 10.3892/or.2017.6140 29251319

[64] Maeda M Hasegawa H Hyodo T Ito S Asano E Yuang H. ARHGAP18, a GTPase-activating protein for RhoA, controls cell shape, spreading, and motility. Mol Biol Cell (2011) 22(20):3840–52. doi: 10.1091/mbc.e11-04-0364 PMC3192863 21865595

[65] Mansouri L Cahill N Gunnarsson R Smedby KE Tjonnfjord E Hjalgrim H. NOTCH1 and SF3B1 mutations can be added to the hierarchical prognostic classification in chronic lymphocytic leukemia. Leukemia (2013) 27(2):512–4. doi: 10.1038/leu.2012.307 23138133

[66] Martinez-Trillos A Pinyol M Navarro A Aymerich M Jares P Juan M. Mutations in TLR/MYD88 pathway identify a subset of young chronic lymphocytic leukemia patients with favorable outcome. Blood (2014) 123(24):3790–6. doi: 10.1182/blood-2013-12-543306 24782504

[67] Parker H Rose-Zerilli MJ Larrayoz M Clifford R Edelmann J Blakemore S. Genomic disruption of the histone methyltransferase SETD2 in chronic lymphocytic leukaemia. Leukemia (2016) 30(11):2179–86. doi: 10.1038/leu.2016.134 PMC5023049 27282254

[68] Walker JS Hing ZA Harrington B Baumhardt J Ozer HG Lehman A. Recurrent XPO1 mutations alter pathogenesis of chronic lymphocytic leukemia. J Hematol Oncol (2021) 14(1):17. doi: 10.1186/s13045-021-01032-2 33451349 PMC7809770

[69] Rossi D Rasi S Spina V Bruscaggin A Monti S Ciardullo C. Integrated mutational and cytogenetic analysis identifies new prognostic subgroups in chronic lymphocytic leukemia. Blood (2013) 121(8):1403–12. doi: 10.1182/blood-2012-09-458265 PMC3578955 23243274

[70] Baliakas P Moysiadis T Hadzidimitriou A Xochelli A Jeromin S Agathangelidis A. Tailored approaches grounded on immunogenetic features for refined prognostication in chronic lymphocytic leukemia. Haematologica (2019) 104(2):360–9. doi: 10.3324/haematol.2018.195032 PMC6355487 30262567

[71] Landau DA Carter SL Stojanov P McKenna A Stevenson K Lawrence MS. Evolution and impact of subclonal mutations in chronic lymphocytic leukemia. Cell (2013) 152(4):714–26. doi: 10.1016/j.cell.2013.01.019 PMC3575604 23415222

[72] Landau DA Sun C Rosebrock D Herman SEM Fein J Sivina M. The evolutionary landscape of chronic lymphocytic leukemia treated with ibrutinib targeted therapy. Nat Commun (2017) 8(1):2185. doi: 10.1038/s41467-017-02329-y 29259203 PMC5736707

[73] Rossi D Khiabanian H Spina V Ciardullo C Bruscaggin A Fama R. Clinical impact of small TP53 mutated subclones in chronic lymphocytic leukemia. Blood (2014) 123(14):2139–47. doi: 10.1182/blood-2013-11-539726 PMC4017291 24501221

[74] Malcikova J Stano-Kozubik K Tichy B Kantorova B Pavlova S Tom N. Detailed analysis of therapy-driven clonal evolution of TP53 mutations in chronic lymphocytic leukemia. Leukemia (2015) 29(4):877–85. doi: 10.1038/leu.2014.297 PMC4396398 25287991

[75] Nadeu F Delgado J Royo C Baumann T Stankovic T Pinyol M. Clinical impact of clonal and subclonal TP53, SF3B1, BIRC3, NOTCH1, and ATM mutations in chronic lymphocytic leukemia. Blood (2016) 127(17):2122–30. doi: 10.1182/blood-2015-07-659144 PMC4912011 26837699

[76] Malcikova J Pavlova S Kunt Vonkova B Radova L Plevova K Kotaskova J. Low-burden TP53 mutations in CLL: clinical impact and clonal evolution within the context of different treatment options. Blood (2021) 138(25):2670–85. doi: 10.1182/blood.2020009530 PMC8703362 33945616

[77] Woyach JA Furman RR Liu TM Ozer HG Zapatka M Ruppert AS. Resistance mechanisms for the bruton's tyrosine kinase inhibitor ibrutinib. N Engl J Med (2014) 370(24):2286–94. doi: 10.1056/NEJMoa1400029 PMC4144824 24869598

[78] Ahn IE Underbayev C Albitar A Herman SE Tian X Maric I. Clonal evolution leading to ibrutinib resistance in chronic lymphocytic leukemia. Blood (2017) 129(11):1469–79. doi: 10.1182/blood-2016-06-719294 PMC5356450 28049639

[79] Woyach JA Ruppert AS Guinn D Lehman A Blachly JS Lozanski A. BTK(C481S)-mediated resistance to ibrutinib in chronic lymphocytic leukemia. J Clin Oncol (2017) 35(13):1437–43. doi: 10.1200/JCO.2016.70.2282 PMC5455463 28418267

[80] Burger JA Landau DA Taylor-Weiner A Bozic I Zhang H Sarosiek K. Clonal evolution in patients with chronic lymphocytic leukaemia developing resistance to BTK inhibition. Nat Commun (2016) 7:11589. doi: 10.1038/ncomms11589 27199251 PMC4876453

[81] Blombery P Anderson MA Gong JN Thijssen R Birkinshaw RW Thompson ER. Acquisition of the recurrent Gly101Val mutation in BCL2 confers resistance to venetoclax in patients with progressive chronic lymphocytic leukemia. Cancer Discovery (2019) 9(3):342–53. doi: 10.1158/2159-8290.CD-18-1119 30514704

[82] Fiorcari S Maffei R Atene CG Mesini N Maccaferri M Leonardi G. Notch2 increases the resistance to venetoclax-induced apoptosis in chronic lymphocytic leukemia b cells by inducing mcl-1. Front Oncol (2021) 11:777587. doi: 10.3389/fonc.2021.777587 35070982 PMC8770925

[83] Jethwa A Hullein J Stolz T Blume C Sellner L Jauch A. Targeted resequencing for analysis of clonal composition of recurrent gene mutations in chronic lymphocytic leukaemia. Br J Haematol (2013) 163(4):496–500. doi: 10.1111/bjh.12539 24032483

[84] Sutton LA Ljungstrom V Enjuanes A Cortese D Skaftason A Tausch E. Comparative analysis of targeted next-generation sequencing panels for the detection of gene mutations in chronic lymphocytic leukemia: an ERIC multi-center study. Haematologica (2021) 106(3):682–91. doi: 10.3324/haematol.2019.234716 PMC7927885 32273480

[85] Stewart JP Gazdova J Darzentas N Wren D Proszek P Fazio G. Validation of the EuroClonality-NGS DNA capture panel as an integrated genomic tool for lymphoproliferative disorders. Blood Adv (2021) 5(16):3188–98. doi: 10.1182/bloodadvances.2020004056 PMC8405189 34424321

[86] Navrkalova V Plevova K Hynst J Pal K Mareckova A Reigl T. LYmphoid NeXt-generation sequencing (LYNX) panel: A comprehensive capture-based sequencing tool for the analysis of prognostic and predictive markers in lymphoid malignancies. J Mol Diagn (2021) 23(8):959–74. doi: 10.1016/j.jmoldx.2021.05.007 34082072

[87] Eichhorst B Robak T Montserrat E Ghia P Niemann CU Kater AP. Chronic lymphocytic leukaemia: ESMO clinical practice guidelines for diagnosis, treatment and follow-up. Ann Oncol (2021) 32(1):23–33. doi: 10.1016/j.annonc.2020.09.019 33091559

[88] Burger JA Barr PM Robak T Owen C Ghia P Tedeschi A. Long-term efficacy and safety of first-line ibrutinib treatment for patients with CLL/SLL: 5 years of follow-up from the phase 3 RESONATE-2 study. Leukemia (2020) 34(3):787–98. doi: 10.1038/s41375-019-0602-x PMC7214263 31628428

[89] Tausch E Schneider C Robrecht S Zhang C Dolnik A Bloehdorn J. Prognostic and predictive impact of genetic markers in patients with CLL treated with obinutuzumab and venetoclax. Blood (2020) 135(26):2402–12. doi: 10.1182/blood.2019004492 32206772

[90] Barr PM Owen C Robak T Tedeschi A Bairey O Burger JA. Up to 8-year follow-up from RESONATE-2: first-line ibrutinib treatment for patients with chronic lymphocytic leukemia. Blood Adv (2022) 6(11):3440–50. doi: 10.1182/bloodadvances.2021006434 PMC9198904 35377947

[91] TamCSAllanJNSiddiqiTKippsTJJacobsROpatS. Fixed-duration ibrutinib plus venetoclax for first-line treatment of CLL: primary analysis of the CAPTIVATE FD cohort. Blood (2022) 139(22):3278–89. doi: 10.1182/blood.2021014488 35196370

[92] Davi F Langerak AW de Septenville AL Kolijn PM Hengeveld PJ Chatzidimitriou A. Immunoglobulin gene analysis in chronic lymphocytic leukemia in the era of next generation sequencing. Leukemia (2020) 34(10):2545–51. doi: 10.1038/s41375-020-0923-9 PMC7515836 32561841

[93] Stamatopoulos K Agathangelidis A Rosenquist R Ghia P. Antigen receptor stereotypy in chronic lymphocytic leukemia. Leukemia (2017) 31(2):282–91. doi: 10.1038/leu.2016.322 27811850

[94] Robbe P Ridout KE Vavoulis DV Dreau H Kinnersley B Denny N. Whole-genome sequencing of chronic lymphocytic leukemia identifies subgroups with distinct biological and clinical features. Nat Genet (2022) 54(11):1675–89. doi: 10.1038/s41588-022-01211-y PMC9649442 36333502

[95] Duncavage EJ Schroeder MC O'Laughlin M Wilson R MacMillan S Bohannon A. Genome sequencing as an alternative to cytogenetic analysis in myeloid cancers. N Engl J Med (2021) 384(10):924–35. doi: 10.1056/NEJMoa2024534 PMC8130455 33704937

[96] Berglund E Barbany G Orsmark-Pietras C Fogelstrand L Abrahamsson J Golovleva I. A study protocol for validation and implementation of whole-genome and -transcriptome sequencing as a comprehensive precision diagnostic test in acute leukemias. Front Med (Lausanne) (2022) 9:842507. doi: 10.3389/fmed.2022.842507 35402448 PMC8987911

[97] Nadeu F Mas-de-Les-Valls R Navarro A Royo R Martin S Villamor N. IgCaller for reconstructing immunoglobulin gene rearrangements and oncogenic translocations from whole-genome sequencing in lymphoid neoplasms. Nat Commun (2020) 11(1):3390. doi: 10.1038/s41467-020-17095-7 32636395 PMC7341758

[98] Oakes CC Seifert M Assenov Y Gu L Przekopowitz M Ruppert AS. DNA Methylation dynamics during b cell maturation underlie a continuum of disease phenotypes in chronic lymphocytic leukemia. Nat Genet (2016) 48(3):253–64. doi: 10.1038/ng.3488 PMC4963005 26780610

[99] Queirós AC Villamor N Clot G Martinez-Trillos A Kulis M Navarro A. A b-cell epigenetic signature defines three biologic subgroups of chronic lymphocytic leukemia with clinical impact. Leukemia (2015) 29(3):598–605. doi: 10.1038/leu.2014.252 25151957

[100] Zhu X He F Zeng H Ling S Chen A Wang Y. Identification of functional cooperative mutations of SETD2 in human acute leukemia. Nat Genet (2014) 46(3):287–93. doi: 10.1038/ng.2894 PMC4440318 24509477

[101] Herbst SA Vesterlund M Helmboldt AJ Jafari R Siavelis I Stahl M. Proteogenomics refines the molecular classification of chronic lymphocytic leukemia. Nat Commun (2022) 13(1):6226. doi: 10.1038/s41467-022-33385-8 36266272 PMC9584885

[102] Argelaguet R Velten B Arnol D Dietrich S Zenz T Marioni JC. Multi-omics factor analysis-a framework for unsupervised integration of multi-omics data sets. Mol Syst Biol (2018) 14(6):e8124. doi: 10.15252/msb.20178124 29925568 PMC6010767

[103] Wang Z Yan H Boysen JC Secreto CR Tschumper RC Ali D. B cell receptor signaling drives APOBEC3 expression *via* direct enhancer regulation in chronic lymphocytic leukemia b cells. Blood Cancer J (2022) 12(7):99. doi: 10.1038/s41408-022-00690-w 35778390 PMC9249768

[104] Nadeu F Royo R Massoni-Badosa R Playa-Albinyana H Garcia-Torre B Duran-Ferrer M. Detection of early seeding of Richter transformation in chronic lymphocytic leukemia. Nat Med (2022) 28(8):1662–71. doi: 10.1038/s41591-022-01927-8 PMC9388377 35953718

[105] ThijssenRTianLAndersonMAFlensburgCJarrattAGarnhamAL. Single-cell multiomics reveal the scale of multilayered adaptations enabling CLL relapse during venetoclax therapy. Blood (2022) 140(20):2127–41. doi: 10.1182/blood.2022016040 35709339

[106] Tamborero D Dienstmann R Rachid MH Boekel J Lopez-Fernandez A Jonsson M. The molecular tumor board portal supports clinical decisions and automated reporting for precision oncology. Nat Cancer (2022) 3(2):251–61. doi: 10.1038/s43018-022-00332-x PMC8882467 35221333

